# A new culture-based method for rapid identification of microorganisms in polymicrobial blood cultures by MALDI-TOF MS

**DOI:** 10.1186/s12866-019-1641-1

**Published:** 2019-11-29

**Authors:** Walter Florio, Susanna Cappellini, Cesira Giordano, Alessandra Vecchione, Emilia Ghelardi, Antonella Lupetti

**Affiliations:** Dipartimento di Ricerca Traslazionale e delle Nuove Tecnologie in Medicina e Chirurgia, Università di Pisa, Via San Zeno 37, 56127 Pisa, Italy

**Keywords:** Bloodstream infection, Polymicrobial blood culture, MALDI-TOF MS, Rapid identification, Selective media

## Abstract

**Background:**

The application of matrix-assisted laser desorption/ionization time of flight (MALDI-TOF) mass spectrometry (MS) to microbial identification has allowed the development of rapid methods for identification of microorganisms directly in positive, blood cultures (BCs). These methods can yield accurate results for monomicrobial BCs, but often fail to identify multiple microorganisms in polymicrobial BCs. The present study was aimed at establishing a rapid and simple method for identification of bacteria and yeast in polymicrobial BCs from patients with bloodstream infection.

**Results:**

The rapid method herein proposed is based on short-term culture in liquid media allowing selective growth of microorganisms recovered from polymicrobial BCs, followed by rapid identification by MALDI-TOF MS. To evaluate the accuracy of this method, 56 polymicrobial BCs were comparatively analyzed with the rapid and routine methods. The results showed concordant identification for both microbial species in 43/50 (86%) BCs containing two different microorganisms, and for two microbial species in six BCs containing more than two different species. Overall, 102/119 (85.7%) microorganisms were concordantly identified by the rapid and routine methods using a cut-off value of 1.700 for valid identification. The mean time to identification after BC positivity was about 4.2 h for streptococci/enterococci, 8.7 h for staphylococci, 11.1 h for Gram-negative bacteria, and 14.4 h for yeast, allowing a significant time saving compared to the routine method.

**Conclusions:**

The proposed method allowed rapid and reliable microbial identification in polymicrobial BCs, and could provide clinicians with timely, useful information to streamline empirical antimicrobial therapy in critically ill patients.

## Background

Bloodstream infections represent a major public health problem being a significant cause of morbidity and mortality worldwide [1–5]. Polymicrobial bloodstream infection is particularly severe, and is commonly associated with dramatically high mortality rates [6–8]. Blood culture (BC), the “gold standard” for the diagnosis of bloodstream infection, requires at least 48–72 h before the results of microbial identification and antimicrobial susceptibility testing can be reported to clinicians. Time to identification may take an additional 18–24 h for polymicrobial BCs as compared to monomicrobial BCs, since sub-culture onto the appropriate solid media may be necessary to isolate all microbial species. To reduce mortality, it is of vital importance to shorten time to diagnosis, and provide clinicians with the necessary information to promptly administer an effective antimicrobial therapy.

The development of new tools and techniques have allowed to reduce time to identification significantly as compared to culture-based methods. In particular, matrix-assisted laser desorption/ionization-time of flight (MALDI-TOF) mass spectrometry (MS) has proved a rapid and reliable method for microbial identification, and a number of recent studies indicate that it could be directly applied to identify microbial pathogens recovered from positive BCs [9–13]. However, microbial identification by MALDI-TOF MS directly in positive BC is intrinsically difficult for polymicrobial BCs, since the peaks of multiple species merge into a single mass spectrum and the results obtained often yield the identification of the predominant microorganism only [14–20].

Direct microbial identification in polymicrobial BCs has proven problematic also when using automated microarray-based assays [21–23].

The aim of the present study was to establish a rapid method for MALDI-TOF MS microbial identification in polymicrobial BCs based on short-term sub-culture in liquid media allowing selective growth of individual species.

## Results

### Evaluation of the accuracy of a rapid method for identification of bacteria and yeast in polymicrobial blood cultures

Overall, 2456 BCs were analyzed, of which 2324 (94.6%) resulted monomicrobial, while 132 (5.4%) revealed polymicrobial growth after Gram staining; among these latter, 56 (42.4%) BCs were analyzed with both the rapid and routine methods since they met the inclusion criteria used in this study, i.e. from each patient, only the first positive BC containing two or more different categories of microorganisms. From the 56 selected BCs, a total of 119 microbial strains were isolated and identified by the routine method; of these, 102 were bacteria, and 17 yeast. In 50 BCs, two distinct microorganisms were identified; five BCs contained three different microbial species, and one BC revealed the presence of four species.

Among the bacterial isolates, 66 were Gram-positive cocci and 36 Gram-negative bacilli. The Gram-positive cocci included: thirty-two coagulase-negative staphylococci (14 *Staphylococcus epidermidis,* ten *Staphylococcus haemolyticus,* three *Staphylococcus hominis,* two *Staphylococcus capitis,* one *Staphylococcus cohnii*, one *Staphylococcus pettenkoferi*, one *Staphylococcus warneri*), two *Staphylococcus aureus*, 26 enterococci (18 *Enterococcus faecalis*, seven *Enterococcus faecium,* one *Enterococcus raffinosus*), four streptococci (three *Streptococcus oralis,* one *Streptococcus pneumoniae*), and two *Granulicatella adiacens*.

The Gram-negative bacilli included 16 *Enterobacteriaceae* (four *Escherichia coli*, four *Klebsiella pneumoniae*, three *Serratia marcescens*, one *Serratia liquefaciens*, one *Serratia urealytica*, one C*itrobacter koseri*, one *Proteus mirabilis*, one member of the *Enterobacter cloacae* complex)*,* and 20 non-fermenting bacteria (12 *Pseudomonas aeruginosa*, six *Acinetobacter baumannii*, one *Stenotrophomonas maltophilia,* one *Burkholderia cepacia*). The identified yeast species included 10 *Candida parapsilosis* and seven *Candida albicans* strains.

In 43 of the 50 (86.0%) BCs in which two microorganisms were present, both were concordantly identified by the rapid and routine methods whereas in the remaining seven BCs (14.0%), only one of the two microorganisms was concordantly identified. In these latter BCs, the second microorganism was not identified by the rapid method (Table 1).

**Table 1:** Identification score of microbial species identified by MALDI-TOF MS after growth in selective liquid media

Sample ID	Not identified by the rapid method	Microbial species identified with score		
		< 1.700	1.700–1.999	≥2.000
BC1				*E. faecalis* *K. pneumoniae*
BC2	*S. capitis*			*E. faecium*
BC3	*S. hominis*			*E. coli*
BC4	*S. haemolyticus*			*K. pneumoniae*
BC5				*C. koseri* *E. faecalis*
BC6				*S. liquefaciens* *E. faecalis*
BC7	*A. baumannii*			*E. faecalis* *P. aeruginosa*
BC8			*A. baumannii*	*E. faecalis*
BC9			*S. haemolyticus*	*P. aeruginosa*
BC10	*S. haemolyticus*		*A. baumannii*	*S. epidermidis*
BC11	*E. faecium*			*K. pneumoniae*
BC12			*S. haemolyticus*	*E. coli*
BC13			*S. capitis*	*E. coli*
BC14	*E. faecalis* *A. baumannii*			*E. faecium* *S. maltophilia*
BC15			*G. adiacens* *S. epidermidis*	
BC16				*C. parapsilosis* *E. faecium*
BC17		*C. albicans*		*E. faecalis*
BC18	*E. faecium*		*C. parapsilosis*	*E. faecalis*
BC19			*S. haemolyticus*	*B. cepacia*
BC20		*C. parapsilosis*		*P. aeruginosa*
BC21	*S. warneri*		*S. oralis* *S. epidermidis*	
BC22		*C. parapsilosis*		*E. cloacae* complex
BC23			*A. baumannii*	*E. faecalis*
BC24				*S. hominis* *C. parapsilosis*
BC25			*S. epidermidis*	*E. faecalis*
BC26	*S. hominis*			*E. coli*
BC27				*S. epidermidis* *C. parapsilosis*
BC28				*E. faecalis* *S. haemolyticus*
BC29				*A. baumannii* *S. marcescens*
BC30				*E. raffinosus* *P. aeruginosa*
BC31				*E. faecalis* *P. aeruginosa*
BC32			*S. haemolyticus*	*S. oralis*
BC33				*E. faecalis* *C. parapsilosis*
BC34				*C. parapsilosis* *S. haemolyticus*
BC35				*S. aureus* *E. faecalis*
BC36				*S. epidermidis* *C. albicans*
BC37				*K. pneumoniae* *C. parapsilosis*
BC38			*S. cohnii*	*C. parapsilosis*
BC39				*P. aeruginosa* *S. pneumoniae*
BC40			*S. epidermidis*	*S. ureilytica*
BC41				*E. faecalis* *P. aeruginosa*
BC42				*S. epidermidis* *E. faecalis*
BC43				*S. epidermidis* *C. albicans*
BC44			*C. albicans*	*E. faecium*
BC45				*S. haemolyticus* *S. marcescens*
BC46				*S. epidermidis* *S. marcescens*
BC47				*P. aeruginosa* *E. faecalis*
BC48				*P. aeruginosa* *S. epidermidis*
BC49	*G. adiacens*			*P. mirabilis*
BC50			*S. pettenkoferi*	*P. aeruginosa*
BC51				*E. faecalis* *P. aeruginosa*
BC52				*S. epidermidis* *P. aeruginosa*
BC53	*S. epidermidis*			*S. aureus* *E. faecium*
BC54			*S. oralis*	*C. albicans*
BC55				*S. epidermidis* *C. albicans*
BC56	*S. haemolyticus*			*C. albicans*

In the six BCs containing three or more microorganisms, two microbes were concordantly identified by the two methods, as reported in Table 1.

In the overall, 105 of the 119 (88.2%) microorganisms were concordantly identified by the rapid and routine methods. In particular, all the 17 (100%) yeasts and 88 out of 102 bacteria (86.3%) were concordantly identified. Among bacteria, Gram-negative bacteria showed an identification rate of 94.4%, enterococci/streptococci 87.1%, and staphylococci 77.1% (Table 2). Among the microorganisms concordantly identified by the two methods, 83/105 (79.0%) had a score ≥ 2.000, 19/105 (18.1%) showed a score between 1.700 and 1.999 and only three (2.9%) a score < 1.700, all the latter three being yeast (Table 1). Considering the high concordance in the identification results obtained, the score threshold for valid species identification by the rapid method could be lowered down to 1.700. Adopting a cut-off value of 2.000, 83/119 (69.7%) microorganisms were identified by the rapid method, whereas lowering the cut-off for valid identification down to 1.700, 102/119 (85.7%) microorganisms could be identified.

**Table 2:** Growth time in selective liquid media, time to identification after blood culture positivity, and number of concordant/correct identifications by the rapid method

Selective culture medium (selected category of microorganisms)	Mean growth time to reach a density of 0.5 McFarland (min–max)	Mean time to identification after BC positivity (min-max)	Number of correct/concordant identifications (%)
Brilliant Green Bile Broth (Gram-negative bacteria)	10.6 h (4.6–20.7 h)	11.1 h (5.1–21.2 h)	34/36 (94.4%)
m-Staphylococcus Broth (staphylococci)	8.2 h (4.9–16.8 h)	8.7 h (5.4–17.3 h)	27/35 (77.1%)
Todd Hewitt Broth w Colistin/Nalidixic acid (streptococci/enterococci)	3.7 h (1.1–22.4 h)	4.2 h (1.6–22.9 h)	27/31 (87.1%)
HB&L Sabouraud (yeast)	13.6 h (8.6–20.6 h)	14.4 h (9.4–21.4 h)	17/17 (100%)

### Time to identification by the rapid method

The mean growth time in selective liquid media to reach a density of 0.5 McFarland was 10.6 h (4.6–20.7 h) in Brilliant Green Bile Broth, 8.2 h (4.9–16.8 h) in m-Staphylococcus Broth, 3.7 h (1.1–22.4 h) in Todd Hewitt Broth w Colistin/Nalidixic acid, and 13.6 h (8.6–20.6 h) in HB&L Sabouraud (Table 2). The longest growth times were observed for *C. parapsilosis* (20.6 h) for yeast, *C. koseri* (20.7 h) for Gram-negative bacteria, *S. capitis* (16.8 h) for staphylococci, and *S. pneumoniae* (22.4 h) for streptococci/enterococci; the shortest growth times in the different groups of microorganisms were found for *C. parapsilosis* (8.6 h), *E. coli* (4.6 h), *S. haemolyticus* (4.9 h), and *E. faecalis* (1.1 h) (data not shown).

After incubation in selective media, cultures were still polymicrobial but enriched of the microbial species positively selected by the growth conditions. In most cases, the microbial load after growth in selective media exceeded 10^9^ CFU/mL for bacteria, and 10^8^ CFU/mL for yeast (data not shown).

Besides the incubation time in the selective media, the total identification time included about 20 min to recover, wash and resuspend bacteria and/or yeast, and 10 min, approximately, for MALDI-TOF MS analysis, for a total of about 30 min. For identification of yeast, sample processing was about 15 min longer than for bacteria due to the necessity of an additional protein extraction step. The mean time to identification after BC positivity by the rapid method for the different microbial categories is reported in Table 2.

## Discussion

Rapid identification of microbial pathogens by MALDI-TOF MS directly from positive BCs is inherently difficult for polymicrobial BCs. In many instances, one of the species is more abundant in the sample biomass, and MALDI-TOF MS analysis yields a single identification confidence score for the predominant microorganism [14–20]. In addition, increased error rates have been documented for polymicrobial in comparison to monomicrobial BCs in the context of direct MALDI-TOF MS [24]. Only a few studies reported that it may be possible to identify multiple microbial species in polymicrobial BCs by MALDI-TOF MS [16, 19, 25, 26]; however, the correct identification of the two different microorganisms was generally limited to a relatively low percentage of polymicrobial cultures [16, 19, 26]. In a recent study, the performance of the Bruker MBT Sepsityper IVD module was evaluated for the direct identification of microorganisms in polymicrobial BCs by MALDI-TOF MS [26]. Using this method, in more than 50% of polymicrobial BCs containing two species, the module correctly identified at least one of the different microorganisms, and in 49/143 (34.3%) samples both microorganisms were identified. A confidence score value threshold ≥1.8 was adopted for species identification, and the module’s ability to detect multiple strains was limited to the identification of maximum two microorganisms.

Impaired performance for the identification of microbial pathogens in polymicrobial BCs have been reported also for molecular methods, such as the Accelerate Pheno system (Accelerate Diagnostics, Inc., Tucson, AZ, USA) [27], the Biofire FilmArray (BioFire Diagnostics, Salt Lake City, UT) [28], *Quick*FISH (AdvanDx, Wolburn, MA) [15], and Verigene (Nanosphere, Northbrook, IL) [15, 28, 29] molecular assays, although in most cases the number of polymicrobial BCs analyzed were very limited; therefore, the accuracy of the analyzed methods could not be determined precisely. Among the tested molecular methods, the best performance has been reported in a study combining the use of the MALDI BioTyper and FilmArray BCID panel compared to the culture-based method [30]. In that study, the MALDI BioTyper allowed to detect 31 polymicrobial BCs for which different Gram stain morphologies were not observed and, in the overall, the FilmArray BCID panel achieved complete identification in 153 (89.5%) out of 171 polymicrobial BCs [30].

The method proposed in the present study is based on short-term culture in selective liquid media of microorganisms recovered from BCs resulting polymicrobial at the Gram stain, followed by MALDI-TOF MS identification. Using this method, in 43 (86%) out of the 50 BCs where two microorganisms were present, both species were concordantly identified by the rapid and routine methods, and in all six BCs containing three or more microbial species, two of them were concordantly identified. In many instances, the lack of identification was due to the presence of two or more microorganisms able to grow in the same selective liquid medium. For example, in sample BC10, *S. haemolyticus* was not identified since its microbial load was much lower than that of *S. epidermidis*, simultaneously present. Analogous observations can be made with regard to samples BC14, BC18, BC21 and BC53. In sample BC49 the lack of identification of *G. adiacens*, concomitantly present together with *P. mirabilis*, might be due to the intrinsic resistance of *Proteus* spp. to colistin, thus resulting in its predominant growth.

Overall, using a cut-off of 2.000, 83/119 (69.7%) microorganisms in polymicrobial BCs were concordantly identified by the rapid method. Adopting a cut-off of 1.700 concordant identification was obtained for 102/119 (85.7%) microorganisms. Of note, identification was correct also for three yeast isolates identified with score values < 1.700. Although score values ≥ 2.000 are generally indicated by the systems’ manufacturers for reliable species identification, it should be considered that this cut-off value has been established for MALDI-TOF MS identification of microorganisms in optimal conditions, i.e. from isolated colonies. However, in some instances, lowering the cut-off value may allow for rapid and correct microbial identification from samples resulting sub-optimal for MALDI-TOF MS analysis. For example, several authors [31–34] observed that cut-off values could be lowered down to some extent without compromising accuracy for MALDI-TOF MS identification of microorganisms recovered from positive BCs, which possibly contain residual blood components affecting the quality of spectra. In the present study, the quality of spectra and, hence, identification scores might be affected by the presence in the sample for MALDI-TOF MS analysis of residual blood components deriving from positive BCs and/or microbial cell material of counter-selected microorganisms after enrichment of the selected microbial species in liquid media. Indeed, lowering the cut-off value for species identification from 2.0 to 1.7 allowed to increase the concordant/correct identification rate of selectively grown microorganisms from 69.7 to 85.7% without affecting accuracy.

The main limitations of this rapid method are represented by i) the exclusion of polymicrobial BCs containing different microbial species that cannot be distinguished by Gram staining, and ii) the lack of identification of different microorganisms able to grow in the same selective medium. Conversely, the strength of the proposed method is that i) time to identification could be remarkably reduced compared to the routine method, which most often requires time consuming sub-culture for isolation of single species onto solid media, ii) it does not require specifically trained personnel, and iii) it is relatively inexpensive.

Despite the observed limitations of the proposed method, it should be considered that in 76.7% of the polymicrobial BCs analyzed, all the microorganisms present were identified by the rapid method.

In recent years, new methods have been proposed to achieve rapid antimicrobial susceptibility testing of bacteria and yeast by MALDI-TOF MS [35–37]. In the next future, further development of antimicrobial susceptibility testing by MALDI-TOF MS-based methods combined with the method herein described could provide relevant information on susceptibility/resistance of microbial pathogens from patients with polymicrobial infection with considerable time saving compared to the routine method.

## Conclusions

In conclusion, the rapid MALDI-TOF MS identification method, herein described, is based on short-term culture in selective liquid media of microorganisms recovered from blood cultures that resulted polymicrobial after Gram staining. This rapid and simple method yielded concordant identification with the routine method by MALDI-TOF MS of both microorganisms present in 43 (86%) out of 50 blood cultures containing two microorganisms, thus providing identification results at least 24 h earlier than the routine method. Although the antimicrobial susceptibility testing cannot be shortened by this method, rapid identification of the infectious agents causing polymicrobial bloodstream infection could be helpful for the clinician to establish an effective empirical antimicrobial therapy for patients who are in particularly critical conditions.

## Methods

### Study population

Blood samples were drawn from patients recovered at the Cisanello Hospital of Pisa (Pisa, Italy). Blood cultures were processed at the Microbiology Unit of the Azienda Ospedaliero-Universitaria Pisana (Pisa, Italy). Samples were inoculated in both aerobic and anaerobic BC bottles (BACTEC Plus/F Aerobic, BACTEC Plus/F Anaerobic, BACTEC Peds Plus/F), and BCs were incubated in the BD BACTEC FX system (Becton Dickinson and Company; Milano, Italia) at 35 +/− 1.5 °C for up to 5 days. From each patient, only the first BC resulting polymicrobial after Gram staining was included in the present study. These included: BCs positive for both Gram-positive and Gram-negative bacteria; BCs containing Gram-positive cocci both in clusters (staphylococci) and in chains (streptococci/enterococci), and BCs in which both Gram-positive and/or Gram-negative bacteria and yeast were detected.

Each BC was analysed using both the routine method and the rapid method established in the present study.

### Routine method for identification of microrganisms in polymicrobial BCs

An aliquot of positive BC resulting polymicrobial at the Gram staining was plated onto appropriate solid media (blood agar, chocolate agar, MacConkey agar, Chapman agar, Schaedler agar for anaerobic cultures only and Sabouraud-dextrose agar for aerobic bottles only). Plates were incubated at 37 °C, 5% CO_2_ for 24–48 h. Then, sub-cultures were streaked onto appropriate solid media, and microbial isolates were identified using a MALDI-TOF Microflex LT Mass Spectrometer (Bruker Daltonics) equipped with a MALDI Biotyper 3.0 software (Bruker Daltonics).

Samples were spotted onto the target plate, and overlaid with 1 μL alpha-cyano-4-hydroxycinnamic acid (HCCA) matrix before MALDI-TOF MS analysis.

### Rapid method for identification of bacteria and yeast from polymicrobial BCs

An 8-mL aliquot of BC resulting polymicrobial after Gram staining was transferred into a BD Serum Separator Tube (BD Vacutainer System, Becton Dickinson). After centrifugation at 2000×g for 10 min, bacteria/yeast were collected from the surface of the silicon layer of the Serum Separator Tube, and suspended in 0.9% saline at final density of 0.65 McFarland (DensiCHECK plus, bioMérieux, l'Etoile, France). A 200-μL aliquot of microbial suspension was used to inoculate the selective broths described below. Serial dilutions of the microbial suspension, in 0.9% saline, were plated onto TSH blood agar (bioMérieux), and the number of colony forming units (CFU) per mL of bacterial suspension was assessed by counting the number of colonies grown on plates after 24 h incubation at 37 °C.

The following selective liquid media were used: Brilliant Green Bile Broth 2% (Biolife Italiana Mascia Brunelli; Milano, Italia), for selective growth of Gram-negative bacteria; Todd Hewitt Broth w Colistin/Nalidixic Acid (Liofilchem; Roseto degli Abruzzi, Teramo, Italia) for selective growth of streptococci and enterococci; for blood cultures containing both streptococci/enterococci and Gram-negative bacteria, 200 μL of 2 μg/mL colistin were added to 2 mL of Todd Hewitt broth to effectively inhibit the growth of Gram-negative bacteria; m-Staphylococcus Broth (Becton Dickinson and Company), containing 7.5% NaCl, for selective cultivation of staphylococci. The selectivity of this medium was enhanced by adding 300 μL of a sterile solution of NaCl at increasing concentrations. The optimal concentration of NaCl was determined experimentally at 12%, as allowing the growth of staphylococci while effectively inhibiting the growth of Gram-negative bacteria, streptococci and enterococci. Finally, HB&L SABOURAUD (Alifax SpA, Polverara, PD, Italy), was used for selective growth of yeast.

The different selective liquid media, dispensed in 2 mL aliquots, were inoculated with 200 μL of bacterial suspension at 0.65 McFarland, and incubated at 37 °C in the Alfred 60/AST instrument (Alifax), allowing a continuous monitoring of bacterial growth, until a turbidity of 0.5 McFarland was reached. Then, an aliquot of 1.5 mL of culture was transferred into a microcentrifuge tube, and centrifuged at 13,000 rpm for 2 min; the supernatant was removed, and the pellet was used for microbial identification by MALDI-TOF MS as described above.

For identification of yeast, a protein extraction was carried out before MALDI-TOF MS analysis. To this purpose, the standard protocol provided by Bruker was slightly modified. The yeast pellet was resuspended in 75% ethanol, followed by centrifugation at 13,000 rpm for 2 min; the supernatant was discarded and the pellet was added 50 μL of 70% formic acid and 50 μL acetonitrile. After vortexing for 2 min, the lysate was centrifuged at 13,000 rpm for 2 min. The supernatant (1 μL), containing the majority of yeast protein, was air-dried and, after addition of the HCCA matrix, used for MALDI-TOF MS analysis as described.

An aliquot of each culture was plated onto TSH blood agar for CFU/mL determination as described above.

## Data Availability

The datasets used and/or analyzed during the current study are available from the corresponding author on reasonable request.

## Abbreviations

AST: Antimicrobial susceptibility testing
BC: Blood culture
BCID: Blood culture identification panel
CFU: Colony forming unit
HCCA: Alpha-cyano-4-hydroxycinnamic acid
MALDI-TOF MS: Matrix-assisted laser desorption/ionization time of flight mass spectrometry
NaCl: Sodium chloride
rpm: Revolutions per minute
TSH: Trypticase soy agar plus 5% sheep blood
